# *Paralichthys olivaceus* MLKL-mediated necroptosis is activated by RIPK1/3 and involved in anti-microbial immunity

**DOI:** 10.3389/fimmu.2024.1348866

**Published:** 2024-01-16

**Authors:** Kangwei Hao, Hang Xu, Shuai Jiang, Li Sun

**Affiliations:** ^1^ CAS and Shandong Province Key Laboratory of Experimental Marine Biology, Institute of Oceanology, CAS Center for Ocean Mega-Science, Chinese Academy of Sciences, Qingdao, China; ^2^ Laboratory for Marine Biology and Biotechnology, Laoshan Laboratory, Qingdao, China; ^3^ College of Earth and Planetary Sciences, University of Chinese Academy of Sciences, Beijing, China

**Keywords:** *Paralichthys olivaceus*, necroptosis, RIPK1, RIPK3, MLKL, immune defense

## Abstract

Necroptosis is a type of proinflammatory programmed necrosis essential for innate immunity. The receptor interacting protein kinases 1/3 (RIPK1/3) and the substrate mixed lineage kinase domain-like protein (MLKL) are core components of the necroptotic axis. The activation and immunological function of necroptosis in fish remain elusive. Herein, we studied the function and activation of RIPK1/3 (PoRIPK1/3) and MLKL (PoMLKL) in teleost *Paralichthys olivaceus*. Bacterial infection increased the expression of RIPK1/3 and MLKL. The N-terminal four-helix bundle (4HB) domain of PoMLKL exhibited necroptosis-inducing activity, and the C-terminal pseudokinase domain exerted auto-inhibitory effect on the 4HB domain. PoRIPK3 was capable of phosphorylating the T360/S361 residues in the PoMLKL C-terminal domain and initiated necroptosis, and this necroptosis-inducing activity was enhanced by PoRIPK1. PoRIPK1/3 interacted with PoMLKL in a manner that depended on the RIP homotypic interaction motif (RHIM), and deletion of RHIM from PoRIPK1/3 led to the dissociation of PoRIPK1/3 with PoMLKL. Inhibition of PoMLKL-mediated necroptosis increased *Edwardsiella tarda* infection in fish cells and tissues, and led to significantly enhanced lethality of the host. Taken together, these results revealed the activation mechanism of PoRIPK1/3-PoMLKL signaling pathway and the immunological function of necroptosis in the immune defense of teleost.

## Introduction

Necroptosis, also known as mixed-lineage kinase domain-like protein (MLKL)-mediated programmed necrosis, plays an important role in the innate immunity (1, 2). Different from apoptosis, which is featured by the activation of caspase-3 and formation of apoptotic bodies without induction of inflammation, necroptosis exhibits distinct biochemical, morphological and immunological characteristics (1–3). When necroptosis occurs, the receptor interacting protein kinase 1 (RIPK1) and 3 (RIPK3) interact with each other via the RIP homotypic interaction motif (RHIM), leading to the phosphorylation and activation of RIPK3 (3, 4). Active RIPK3 then recruits and phosphorylates the necroptosis executioner, MLKL. Phosphorylation unleashes the N-terminal (NT) four-helix bundle (4HB) domain of MLKL from the C-terminal (CT) pseudokinase (PsKD) domain (5, 6). This conformation change switches MLKL from the inactive state to the active state, allowing MLKL translocation, oligomerization, and forming transmembrane channels on the cytoplasmic membrane (7, 8). The membrane permeabilization disrupts cellular ion homeostasis and causes osmotic cell swelling and cytoplasmic membrane lysis, resulting in massive release of cytoplasmic contents and induction of inflammatory immune response (6, 9, 10).

Increasing studies reported the critical role of necroptosis in infection induced immune responses. For instance, *Salmonella enterica* infection activated the RIPK1/3-MLKL signaling pathway in mouse and induced necroptosis that led to rapid host death (11). Infection of *Listeria monocytogenes* triggered necroptosis in mouse Kupffer cells, which recruited monocytes to the damaged tissues and provoked inflammatory immune response (12). By contrast, blockage of the necroptotic signaling pathway in mouse protects cells from infection-induced cell death (13). In herpes simplex virus-1 infected patients, inherited RIPK3 deficiency prohibited the phosphorylation of MLKL, and thus impaired the necroptosis-dependent immune defense against virus (14).

Although necroptosis has been proved to be essential for host immunity in mammals such as human and mice, the necroptotic signaling pathway is poorly conserved during evolution (15). Genome sequencing showed that in teleost, some species, such as zebrafish *Danio rerio*, lack MLKL homolog. In stickleback *Gasterosteus aculeatus*, MLKL homolog was identified, whereas the N-terminal region of stickleback MLKL was unable to induce cell death (16, 17). The necroptotic activity of MLKL in fish thus remains to be elucidated. RIPK1 and RIPK3 are ubiquitously present in teleost. In half-smooth tongue sole *Cynoglossus semilaevis*, RIPK3 was reported to exhibit pro-apoptotic and pro-necroptotic effect (18). In zebrafish, due to the lack of MLKL homolog, RIPK1/3 were reported to be involved in anti-microbial immunity probably in a necroptosis-independent manner (19, 20).

In this study, by searching the teleost genomic database, we found that Japanese flounder *Paralichthys olivaceus* possessed RIPK1/3 (designated PoRIPK1/3) and MLKL (designated PoMLKL) homologs. The aim of this study was to examine the necroptosis mediated by flounder PoRIPK1/3 and MLKL. We determined the expression of PoRIPK1/3 and PoMLKL during bacterial infection, examined the phosphorylation and activation of PoMLKL by PoRIPK1/3, and revealed the role of necroptosis in the teleost immune defense against bacterial infection. Collectively, these results provide important insights into the regulation and function of necroptosis in teleost.

## Results

### Structural and expression characteristics of PoMLKL and PoRIPK1/3

**Figure 1:**
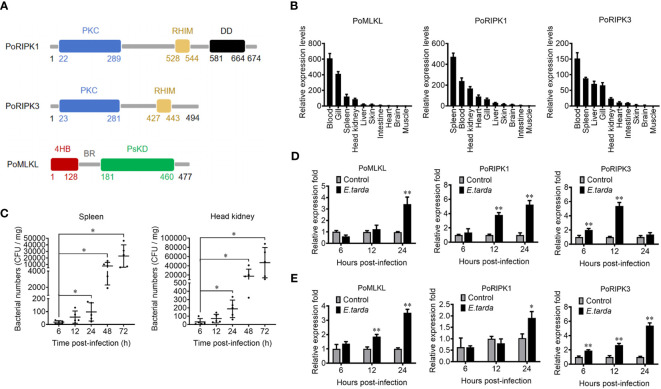
The domain structure and tissue expression of PoMLKL, PoRIPK1, and PoRIPK3. **(A)** Domain analysis of PoMLKL, PoRIPK1, and PoRIPK3. 4HB, four-helical bundle domain; BR, brace region; PsKD, pseudokinase domain; PKC, protein kinase C domain; RHIM, RIP homotypic interaction motif; DD, death domain. **(B)** The relative expression levels of PoMLKL, PoRIPK1 and PoRIPK3 in different tissues of *Paralichthys olivaceus* were determined by quantitative real time-PCR (qRT-PCR). The expression levels are presented relative to that in muscle. **(C)** *P. olivaceus* were infected with *Edwardsiella tarda*, and the bacterial numbers in spleen and head kidney were determined at different time points. Values are the means ± SD. n = 5. *P < 0.05. **(D, E)** *P. olivaceus* were infected with or without (control) *E. tarda*, and the expression of PoMLKL, PoRIPK1 and PoRIPK3 in spleen **(D)** and head kidney **(E)** was determined by qRT-PCR at different time points. In each case, the expression level of the control fish was set as 1. Values are the means ± SD. n = 3. *P < 0.05, **P < 0.01.

MLKL (designated PoMLKL) and RIPK1/3 (designated PoRIPK1/3) homologs were identified in teleost *Paralichthys olivaceus*. PoMLKL contains an N-terminal four-helix bundle (4HB) domain and a C-terminal pseudokinase domain (PsKD) connected by an interdomain brace region (BR) (**Figure 1A**). The protein architecture of PoMLKL is similar to that of human and mouse MLKL (21). PoRIPK1 and PoRIPK3 exhibit similar protein architectures, both harboring an N-terminal protein kinase C (PKC) domain and a C-terminal RIP homotypic interaction motif (RHIM) (**Figure 1A**). PoMLKL, PoRIPK1 and PoRIPK3 were expressed abundantly in immune related tissues, including spleen, blood, head kidney and gill, in *P. olivaceus* (**Figure 1B**). When the fish were infected with *Edwardsiella tarda*, a serious pathogen causing haemorrhagic septicaemia and gastroenteritis in fish, increasing bacterial load was detected in spleen and head kidney in a time dependent manner (**Figure 1C**). In the infected fish, the expressions of PoMLKL, PoRIPK1, and PoRIPK3 in spleen were significantly upregulated at 24 hpi, 12 hpi and 24 hpi, 6 hpi and 12 hpi, respectively (**Figure 1D**). The expressions of PoMLKL, PoRIPK1, and PoRIPK3 in head kidney were significantly upregulated at 12 hpi and 24 hpi, 24 hpi, and 6 hpi to 24 hpi, respectively (**Figure 1E**).

### PoMLKL and its truncates exhibit necroptotic activity and bactericidal activity, respectively

**Figure 2:**
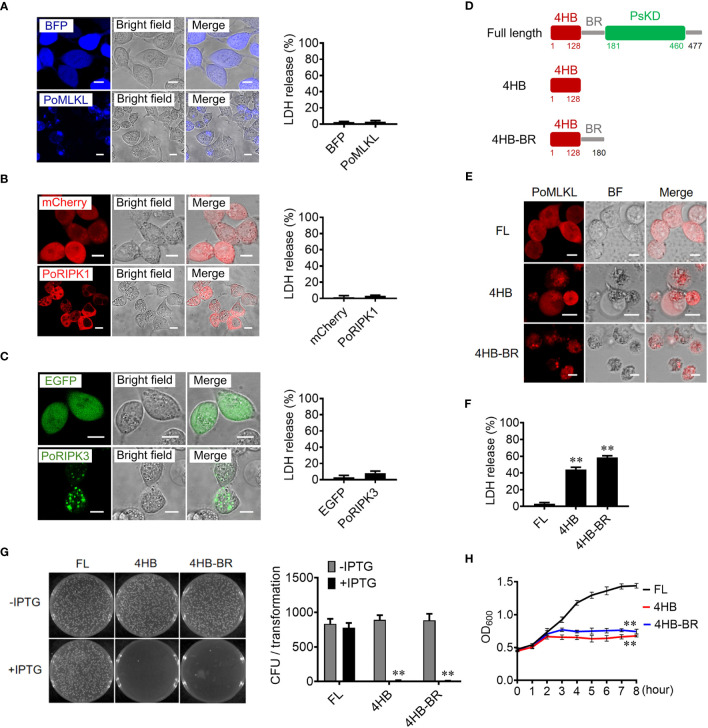
The necroptotic and antibacterial activity of PoMLKL. **(A–C)** HEK293T cells were transfected with the vectors expressing BFP-tagged PoMLKL **(A)**, mCherry-tagged PoRIPK1 **(B)**, and EGFP-tagged PoRIPK3 **(C)**. The control cells were transfected with the vectors expressing BFP **(A)**, mCherry **(B)** or EGFP **(C)**. The expression and subcellular distribution of the proteins were observed with a confocal microscope. Scale bar, 10 μm. The release of LDH from the above cells was determined. Values are the means ± SD. n = 3. **(D)** Schematic presentation of PoMLKL full length (FL) and truncates. 4HB, four-helical bundle domain; BR, brace region; PsKD, pseudokinase domain. **(E, F)** HEK293T cells were transfected with mCherry-tagged PoMLKL FL or truncate (4HB or 4HB-BR), and cell death was observed with a confocal microscope **(E)**. Scale bar, 10 μm. The LDH release was measured and statistically calculated **(F)**. Values are the means ± SD. n = 3. **P < 0.01. **(G)** *Escherichia coli* carrying PoMLKL-FL, 4HB, and 4HB-BR genes were grown in LB agar plates in the presence or absence of the inducer IPTG overnight. Bacterial growth was then observed (left panel) and the colony forming units (CFUs) were calculated (right panel). Values are the means ± SD. n = 3. **P < 0.01. **(H)** *E. coli* carrying PoMLKL-FL, 4HB, and 4HB-BR genes were grown in LB medium supplemented with or without IPTG for different hours. The bacterial growth was monitored by measuring OD600. Values are the means ± SD. n = 3. **P < 0.01.

When ectopically expressed in HEK293T cells, PoRIPK1 distributed evenly in the cytoplasm, while PoRIPK3 and PoMLKL formed small puncta and larger aggregates, respectively, in the cytoplasm (**Figures 2A–C**). Compared with the control cells, the cells expressing PoRIPK1, PoRIPK3, and PoMLKL showed normal morphologies with little LDH release (**Figures 2A–C**). To examine whether PoMLKL possessed necroptosis-inducing capacity, two PoMLKL truncates were constructed, one consisting of the 4HB domain and the other consisting of 4HB plus the BR region (4HB-BR) (**Figure 2D**). Compared with the cells expressing full length (FL) PoMLKL, the cells expressing 4HB or 4HB-BR underwent necroptosis, accompanying with osmotic swelling and massive release of LDH (**Figures 2E, F**). When expressed in *Escherichia coli*, 4HB and 4HB-BR inhibited bacterial growth, whereas PoMLKL had no effect on bacterial growth (**Figures 2G, H**).

### PoRIPK3 is required for PoMLKL-mediated necroptosis

**Figure 3:**
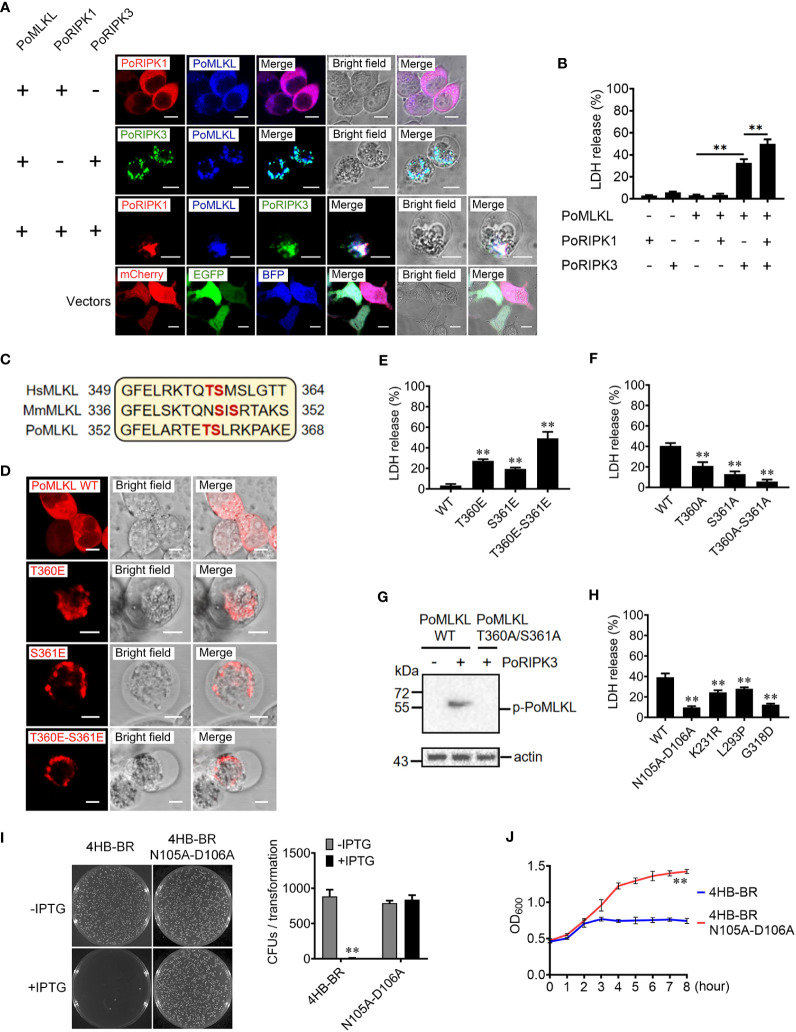
The effects of PoRIPK1 and PoRIPK3 on PoMLKL activation. **(A)** HEK293T cells were co-transfected with backbone vectors or vectors expressing PoMLKL, PoRIPK1 and PoRIPK3. The subcellular distribution of these proteins was observed with a confocal microscope. Scale bar, 10 μm. **(B)** The release of LDH from the above transfected cells was determined. Values are the means ± SD. n = 3. **P < 0.01. **(C)** Sequence analysis of the region containing the phosphorylation residues in human MLKL (HsMLKL), mouse MLKL (MmMLKL), and PoMLKL. **(D)** HEK293T cells were transfected with vectors expressing PoMLKL wild type (WT) or mutants, and then observed with a confocal microscope. Scale bar, 10 μm. **(E)** The release of LDH from the above transfected cells was determined. Values are the means ± SD. n = 3. **P < 0.01. **(F, G)** PoMLKL WT or mutant was expressed in HEK293T cells in the absence or presence of PoRIPK3. The release of LDH was determined **(F)** and the phosphorylation of PoMLKL was detected by immunoblotting **(G)**. Values in **(F)** are the means ± SD. n = 3. **P < 0.01. **(H)** HEK293T cells were co-transfected with PoMLKL mutants and PoRIPK3, and the release of LDH was determined. Values are the means ± SD. n = 3. **P < 0.01. **(I)** *E. coli* cells expressing PoMLKL 4HB-BR or its N105A-D106A mutant were grown in the presence or absence of IPTG overnight (left panel), and the colony forming units (CFUs) were statistically calculated (right panel). Values are the means ± SD. n = 3. **P < 0.01. **(J)** *E. coli* cells expressing PoMLKL 4HB-BR or its N105A-D106A mutant were grown in the presence or absence of IPTG for different hours. The bacterial growth was monitored by measuring OD600. Values are the means ± SD. n = 3. **P < 0.01.

To determine the activation of PoMLKL-mediated necroptosis, we co-expressed PoMLKL with PoRIPK1 and/or PoRIPK3 in HEK293T cells. Confocal microscopic analysis showed that co-expression of PoRIPK1 and PoMLKL did not lead to cell death, but co-expression of PoRIPK3 and PoMLKL induced necroptosis and massive release of LDH (**Figures 3A, B**). PoMLKL was colocalized with PoRIPK3 but not with PoRIPK1 (**Figure 3A**). When PoMLKL, PoRIPK1, and PoRIPK3 were co-expressed, PoRIPK1 colocalized with PoRIPK3 and PoMLKL, and necroptosis occurred with higher LDH release (**Figures 3A, B**). The sequence alignment showed that there exist two potential phosphorylation residues, T360 and S361, in PoMLKL, which correspond to the phosphorylation residues in human MLKL (HsMLKL) (T357 and S358) and mouse MLKL (MmMLKL) (S345 and S347) (**Figure 3C**). Previous studies reported that phosphorylation of these residues in HsMLKL and MmMLKL incurred the conformation switch from inert to active state (22). In order to determine the role of these residues in PoMLKL-mediated necroptosis, we mutated either or both residues to Glu, which mimicked the negatively charged phosphate group. When expressed in HEK293T cells, PoMLKL bearing T360E or S361E mutation induced necroptosis and LDH release, and PoMLKL bearing T360E-S361E double mutation induced necroptosis and more LDH release (**Figures 3D, E**). We next mutated PoMLKL T360 and/or S361 to Ala, which could not be phosphorylated by PoRIPK3. When the mutant T360A or S361A was co-expressed with PoRIPK3, LDH release was significantly decreased compared with cells co-expressing PoMLKL and PoRIPK3 (**Figure 3F**). When the double mutant T360A-S361A was co-expressed with PoRIPK3, little LDH release was observed (**Figure 3F**). Furthermore, unlike PoMLKL, which was phosphorylated by the co-expressed PoRIPK3, the T360A-S361A mutant was not phosphorylated by PoRIPK3 (**Figure 3G**). Besides T360 and S361, other residues of PoMLKL, including N105 and D106 in the 4HB domain and L293, K231, and G318 in the CT domain, were also mutated to examine their effect on necroptosis. When co-expressed with PoRIPK3, the mutants of L293P, K231R, G318D, and N105A-D106A induced significantly reduced LDH release (**Figure 3H**). Moreover, the 4HB-BR domain bearing N105A-D106A mutation lost the ability to kill bacteria (**Figures 3I, J**).

### RHIM is required for PoRIPK1/3 activation of PoMLKL

**Figure 4:**
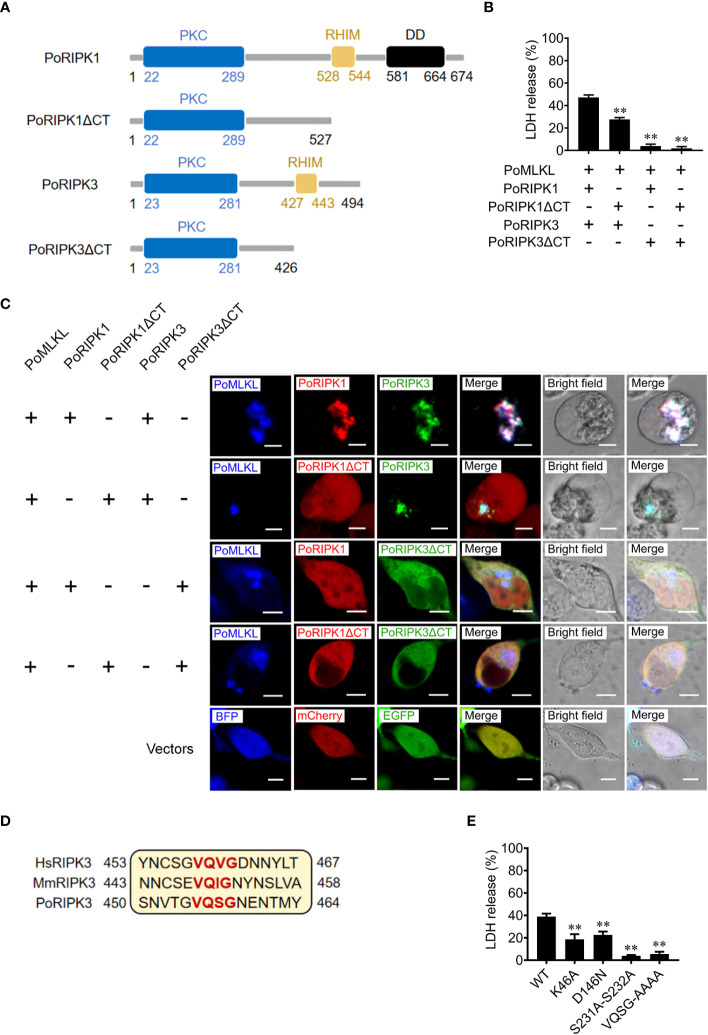
Key residues of PoRIPK1/3 involved in PoMLKL activation. **(A)** Schematic representation of PoRIPK1/3 and truncated mutants. PKC, protein kinase C domain; RHIM, RIP homotypic interaction motif; DD, death domain. **(B, C)** HEK293T cells were co-transfected with vectors expressing PoMLKL plus PoRIPK1/3 or truncated mutants. **(B)** The release of LDH from the transfected cells was determined. Values are the means ± SD. n = 3. **P < 0.01. **(C)** The above transfected cells were observed with a confocal microscope. Scale bars, 10 μm. **(D)** Sequence alignment of the motifs critical for oligomerization in human, mouse, and flounder RIPK3 (HsRIPK3, MmRIPK3, and PoMLKL, respectively). The conserved tetrapeptides are marked red. **(E)** HEK293T cells were co-transfected with vectors expressing PoMLKL plus PoRIPK3 wild type (WT) or mutants. The release of LDH was determined. Values are the means ± SD. n = 3. **P < 0.01.

To explore the role of RHIM in PoRIPK1/3 activation of PoMLKL, the mutants PoRIPK1ΔCT and PoRIPK3ΔCT were constructed, which lacked the CT region containing RHIM (**Figure 4A**). When PoRIPK1ΔCT was co-expressed with PoRIPK3 and PoMLKL in HEK293T cells, PoRIPK1ΔCT was not able to colocalize with PoRIPK3 and PoMLKL, but PoRIPK1ΔCT had no effect on PoRIPK3 colocalization with PoMLKL and necroptosis induction (**Figures 4B, C**). It should be noted that, compared with cells co-expressing PoRIPK1, PoRIPK3, and PoMLKL, cells co-expressing PoRIPK1ΔCT, PoRIPK3, and PoMLKL released significantly lower LDH (**Figure 4B**). When co-expressed with PoRIPK3ΔCT, neither PoRIPK1 nor PoMLKL could interact with PoRIPK3ΔCT, and no cell death or LDH release was observed (**Figures 4B, C**). The tetrapeptide motif of VQVG (human) and VQIG (mouse) in the RIPK3 RHIM domain are proved to be essential for homo-oligomerization and MLKL activation (3, 23, 24). Mutation of the corresponding tetrapeptide (VQSG) in PoRIPK3 to AAAA significantly decreased the induction of PoMLKL-mediated necroptosis (**Figures 4D, E**). In addition to VQSG, the importance of four residues (K46, D146, S231 and S232) in the N-terminal PKC domain of PoRIPK3 was also analyzed. Compared with PoRIPK3, the mutants of K46A, D146N, and S231A-S232A exhibited attenuated ability to activate PoMLKL, indicated by significantly decreased LDH release (**Figure 4E**).

### MLKL-mediated necroptosis is involved in bacterial clearance

**Figure 5:**
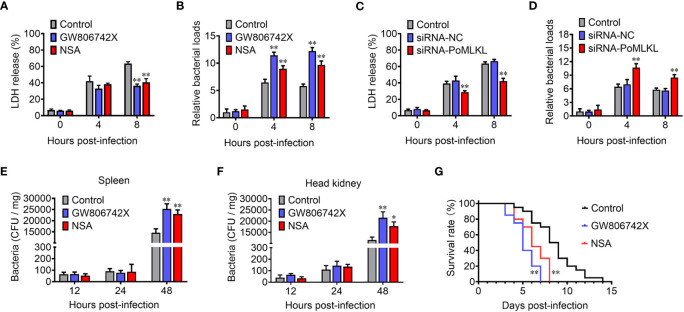
PoMLKL is critical for the anti-infectious immunity. **(A, B)** Flounder FG-9307 cells were pretreated with or without (control) MLKL inhibitor GW806742X or NSA, and then infected with *Edwardsiella tarda* for different hours. The release of LDH from the infected cells **(A)** and the intracellular bacterial numbers **(B)** were determined. **(C, D)** FG-9307 cells were treated with or without (control) siRNA-MLKL (PoMLKL specific siRNA) or siRNA-NC (negative control siRNA), and then infected with *E. tarda* for different hours. The release of LDH from the infected cells **(C)** and the intracellular bacterial numbers **(D)** were determined. **(E–G)** Fishes were infected with *E. tarda* in the presence or absence (control) of GW806742X or NSA. Bacterial numbers in spleen **(E)** and head kidney **(F)** were determined at different time points, and the fish survival was statistically calculated with log rank test **(G)**. For **(A–F)**, values are the means ± SD. n = 3, *P < 0.05, **P < 0.01. For panel G, n = 20, **P < 0.01.

In order to investigate the effect of necroptosis on bacterial infection, MLKL-mediated necroptosis in flounder cells (FG-9307) was inhibited by the MLKL specific inhibitor GW806742X or NSA (**Supplementary Figure S1**). In the presence of the inhibitor, *E. tarda* infection induced cell death was significantly decreased, and the intracellular bacterial load was significantly increased (**Figures 5A, B**). We further knocked down the expression of PoMLKL by using siRNA (**Supplementary Figure S2**), and found that *E. tarda* infection-induced cell death significantly decreased, and the intracellular bacterial number was significantly increased (**Figures 5C, D**). When flounder were infected with *E. tarda* in the presence of MLKL inhibitors, the bacterial load in spleen and head kidney significantly increased, and host mortality occurred early (**Figures 5E–G**). These results collectively indicated that necroptosis exerted anti-microbial effects in fish immunity.

## Discussion

RIPK1/3 and MLKL are the key components of the necroptotic machinery (25–27). In human and mouse, RIPK1 and RIPK3 interact to form necrosome, which in turn phosphorylates MLKL and induces necroptosis (4, 28, 29). Although necroptosis is proved to be of great importance to the innate immunity in mammals, the necroptotic signaling axis is poorly conserved (30, 31). In teleost, RIPK1 and RIPK3 are ubiquitously existed, however, the necroptosis executioner MLKL homolog is absent in some species, including zebrafish *D. rerio*, the pufferfish *Takifugu rubripes* and *Tetraodon nigroviridis* (15, 32). In the present study, we found that there existed the core necroptotic signaling elements, PoRIPK1, PoRIPK3, and PoMLKL, in teleost *P. olivaceus*, and all these three proteins exhibit conserved protein architecture to their mammalian counterparts, suggesting the existence of necroptotic signaling pathway in *P. olivaceus*.

MLKL exerts necroptotic-inducing activity through its N-terminal domain (33, 34). In the present study, although expression of full length PoMLKL was incapable of inducing cell death, the removal of C-terminal PsKD domain was sufficient to activate PoMLKL-mediated necroptosis. The importance of the MLKL N-terminal 4HB domain in executing necroptosis has been well studied in human. Compared with the full length or the C-terminal domain, human MLKL 4HB domain is capable of inducing necroptosis (8, 9). The structural integrity of the N-terminus is essential to the pore-forming capacity of MLKL. Truncated human MLKL 4HB-BR domain with N-terminus deletion (23-180 aa) failed to form oligomerization and was unable to induce necroptosis (8). In this study, we identified functional residues of PoMLKL involved in necroptosis-inducing. We found that PoMLKL N105A-D106A mutant was defective to induce necroptosis in the presence of PoRIPK3. This observation was consistent with that of mouse MLKL mutant R105A-D106A, which was unable to translocate on the cytoplasmic membrane and oligomerize to form transmembrane channels (16). Besides the functional residues (N105 and D106) in the PoMLKL N-terminal domain, we also identified three residues, L293, K231 and G318, in the C-terminal PsKD domain required for inducing necroptosis. PoMLKL L293 corresponds to mouse MLKL L280, mutation of which was reported to lead to inability of mouse MLKL in inducing necroptosis (35). K231 is conserved in human (K230) and mouse (K219) MLKL, ubiquitylation of K219 was reported to enhance mouse MLKL membrane perforation (36). PoMLKL G318 corresponds to human MLKL G316, and mutation of G316 inhibited the RIPK3-dependent phosphorylation of human MLKL (37).

Although both PoRIPK1 and PoRIPK3 contain the N-terminal PKC domain, only PoRIPK3 was required for PoMLKL phosphorylation and inducing necroptosis, suggesting that PoRIPK3 was sufficient to activate PoMLKL. Indeed, we found that PoRIPK3 could phosphorylate PoMLKL at residues T360 and S361, which are conserved phosphorylation sites in human and mouse MLKL. Consistent with the observation in human and mouse MLKL, PoMLKL mutant T360A-S361A was not phosphorylated by PoRIPK3 and thus incapable of inducing necroptosis. By contrast, the T360E and S361E mutants were constitutively active and able to induce necroptosis. These results indicated that MLKL activation by RIPK3-mediated phosphorylation is conserved in fish.

Necroptosis is a highly proinflammatory programmed cell death that is essential to the immune defense against infections (38). The cell lysis induced by active MLKL releases massive intracellular damage-associated molecular patterns, such as interleukin-1α, ATP, genomic and mitochondrial DNA, which lead to inflammation and recruitment of immune cells to the sites of infection (39, 40). In the present study, we revealed the PoRIPK1/3-dependent PoMLKL activation, which induced cell lysis and massive release of cytoplasmic contents. Bacterial infection increased the expression level of PoRIPK1, PoRIPK3, and PoMLKL in *P. olivaceus*, while inhibition of PoMLKL activation impaired the immune defense of the host, suggesting conserved immune function of necroptosis during evolution. In addition to the perforation of host cells, MLKL also shows lipophilic binding preference to cardiolipin, an essential component of bacterial membrane, and is able to lyse the cardiolipin-containing liposomes (5, 8). Active MLKL was found to exert direct bactericidal activity. For instance, *Listeria monocytogenes* infection induced activation of RIPK3-MLKL signaling axis in human cells, and the active MLKL directly bound to the bacteria and inhibited the bacterial replication (41). In our present study, we observed the direct bactericidal activity of PoMLKL, indicating the conserved bactericidal activity of MLKL during evolution. A recent study reported a different mechanism of MLKL in the anti-microbial immune defense. Ubiquitination of phosphorylated human MLKL switched MLKL translocation to endosomes, and enhanced bacterial clearance by promoting the trafficking *Yersinia enterocolitica* to lysosomes (42). MLKL-mediated necroptosis therefore has multifaceted roles in the anti-microbial immunity.

## Materials and methods

### Animal

Clinically healthy *Paralichthys olivaceus* were obtained from a fish farm. The fish were kept at 20°C in aerated seawater as reported previously (43). To obtain tissue samples, the fish were put to death using an excessive amount of tricaine methane sulfonate (Sigma, St. Louis, MO). All live animal experiments were approved by the Ethics Committee at the Institute of Oceanology, Chinese Academy of Sciences.

### Gene cloning and sequence mutagenesis

The GenBank ID of *PoRIPK1*, *PoRIPK3*, and *PoMLKL* are 109624097, 109645932 and 10964593, respectively. PCR was employed to amplify the codon-optimized protein coding sequences (CDS) of PoMLKL, PoRIPK1, and PoRIPK3. Site-directed mutations of PoRIPK3 K46A, D146N, S231N/S232N and ^455^VQSG^458^−^455^AAAA^458^, and PoMLKL N105A-D106A, K231R, L293P, G318D, S360A, S361A, S360A-S361A, S360E, S361E, and S360E-S361E were performed using the Hieff Mut Site-Directed Mutagenesis Kit (Yeasen, Shanghai, China). The accuracy of the mutations was confirmed through sequencing analysis. The primers used are listed in **Supplementary Table S1**.

### Immunoblotting

To detect the expression or phosphorylation of PoMLKL, immunoblotting was conducted as reported previously (44, 45). In brief, protein samples were subjected to SDS-PAGE and subsequently transferred onto nitrocellulose membranes. Following a 5% skim milk blocking step, the membranes were probed with primary antibody and then with a secondary antibody labeled with horseradish peroxidase (HRP). The primary antibodies utilized were mouse antibodies targeting HsMLKL (phospho S358) (1:3000 dilution) and β-actin (1:5000 dilution). The secondary antibody was goat anti-mouse IgG-HRP antibody (1:5000 dilution). All antibodies were obtained from Abcam in Cambridge, MA, USA.

### Cell culture and transfection

HEK293T cells (American Type Culture Collection) were cultivated in Dulbecco’s modified Eagle’s medium supplemented with 10% fetal bovine serum (Gibco, Renfrewshire, UK) at 37°C in a 5% CO_2_ environment. *Edwardsiella tarda* were cultured in Luria Bertani broth (LB) medium with shaking at 28°C (180 rpm). Transient transfection of plasmid into HEK293T cells was performed using Polyjet Transfection Reagent (Signagen, USA). For gene overexpression experiments, pmCherry-N1 vectors (Clontech, Mountain View, CA, USA) expressing PoMLKL variants, PoRIPK1, or PoRIPK1ΔCT were constructed, pEGFP-C1 vectors (Clontech, Mountain View, CA, USA) expressing PoRIPK3 variants were constructed, and pCMV-N-BFP vector (Beyotime, CN) expressing PoMLKL was constructed. HEK293T cells were transfected with these constructs, and the control cells were transfected with the backbone vectors. All plasmids were extracted using endotoxin-free plasmid kit (Sparkjade Biotechnology Co. Ltd., Shandong, China).

### Lactate dehydrogenase assay

To assess cell death, the activity of lactate dehydrogenase (LDH) released into the cell culture supernatant was measured using the CytoTox96 LDH release kit (Promega, Leiden, Netherlands) as reported previously (46). The percentage of cytotoxicity was calculated using the formula: percent cytotoxicity = 100 × (experimental sample - culture medium background)/(maximum LDH release - culture medium background).

### Microscopy

Microscopy display was performed as previously reported (47). To examine cell morphology, the cells were plated on 24-well plate (Costar, Corning, NY, USA) at about 60% confluency and subjected to the indicated treatment. The bright-field and fluorescent views of the cells were recorded using a Carl Zeiss LSM 710 confocal microscope (Carl Zeiss, Jena, Germany).

### Quantitative real time RT-PCR

qRT-PCR was performed as reported previously (48). To explore of gene expression in fish tissue, aseptic collection of tissues (blood, gill, spleen, head kidney, liver, skin, intestine, heart, brain and muscle) was performed with three fish specimens. Total RNA was extracted from tissues using Trizol reagent (Invitrogen, Carlsbad, CA, USA). cDNA synthesis was carried out using the First Strand cDNA Synthesis Kit (ToYoBo, Japan) as per the manufacturer’s instructions. qRT-PCR was conducted on an Eppendorf Mastercycler (Eppendorf, Hamburg, Germany) with the SYBR ExScript qRT-PCR Kit (Vazyme Biotech Co. Ltd., Nanjing, China). To investigate gene expression in *E. tarda*-infected fish, *P. olivaceus* were randomly divided into two groups (35 fish per group) and intramuscularly injected with *E. tarda* (1 × 10^6^ CFU/fish) or PBS. Tissue samples were collected at 6, 12, and 24 h post-infection (hpi) (9 fish/each time point) and used for qRT-PCR as above. The mRNA levels of the target genes were normalized to the expression of β-actin.

### RNA interference

The small interfering RNA (siRNA) sequence targeting PoMLKL (siRNA-PoMLKL) was provided by Sangon (Shanghai, China). The scrambled control (siRNA-NC) served as a negative control and was obtained from the same company. SiRNA was introduced into FG-9307 cells using RNAiMAX transfection reagent (Invitrogen, USA). The interference efficiency was determined by qRT-PCR as above.

### *In vitro* infection

FG-9307 cells were plated on 96-well plate (Costar, Corning, NY, USA) at about 60% confluency. *E. tarda* was cultured in LB broth at 28°C to OD_600_ 0.8. The bacteria were washed with PBS and resuspended in PBS. FG-9307 cells were pretreated with MLKL inhibitors (1 μM GW806742X or 1 μM NSA) (MedChem Express, NJ, USA) for 6 h, or transfected with siRNA as described above for 24 h. The cells were then infected with or without (control) *E. tarda* (MOI = 10:1) at room temperature for 6 h. Cell death was determined by LDH release assay. To determine bacterial load, cells were lysed with Triton X-100 solution (final concentration of 1%), and the lysate was applied to LB plates containing 50 μg/ml tetracycline. The plate was incubated at 28°C for 24 h, and the number of colonies on the plate was counted.

### Bacterial dissemination in fish tissues

A total of 90 P*. olivaceus* specimens were randomly divided into three groups, with 30 fish in each group. The groups received intraperitoneal injections of either 20 mg/kg NSA (MedChem Express, NJ, USA) or 20 mg/kg GW806742X (MedChem Express, NJ, USA), while the control group was injected with equal volume of sterile PBS. At 8 h after injection, all fish were intramuscularly inoculated with 5 × 10^5^ CFU of E. tarda. At 12, 24, and 48 hpi, head kidney and spleen samples were collected (nine fish/each time point). For each time point, the tissues of three fish were mixed and homogenized in sterile PBS. The homogenates were diluted in PBS in a stepwise manner and then spread on LB agar plates. After incubating the plates at 28°C for 18 h, the number of colonies on the plate was counted.

### Fish survival after bacterial challenge

A total of 60 P*. olivaceus* specimens were divided into three groups (20 fish each group). The three groups were intraperitoneally injected with NSA, GW806742X, or PBS as above. At 8 h after injection, all groups were infected with *E. tarda* as above. The fish were then monitored daily for mortality.

### Bactericidal assay

*E. coli* Transetta (DE3) was transformed with pET-30a expressing PoMLKL variants (FL, 4HB, 4HB-BR, 4HB-BR, and N105A-D106A). The transformants were grown in LB medium supplemented with 50 mg/ml kanamycin until OD_600_ 0.4-0.6. The cells were then diluted and cultured on LB agar plates containing 50 mg/ml kanamycin with or without 0.2 mM IPTG. After overnight incubation at 37°C, the colony-forming units (CFU) on the plates were counted and statistically analyzed. The transformants were also cultured in LB medium to OD_600_ 0.5, and then 0.2 mM IPTG was added to the culture. Bacterial growth was recorded every hour.

### Statistical analysis

The two-sample Student *t* test was used for comparisons between groups. Log-rank was used for the analysis of fish survival. Statistical analysis was performed with GraphPad Prism 7 software. (https://www.graphpad.com/). Statistical significance was defined as *P* < 0.05.

## Data availability statement

The original contributions presented in the study are included in the article/**Supplementary Material**. Further inquiries can be directed to the corresponding authors.

## Ethics statement

The animal study was approved by Institute of Oceanology Chinese Academy of Sciences. The study was conducted in accordance with the local legislation and institutional requirements.

## Author contributions

KH: Writing – original draft, Data curation, Formal analysis, Investigation, Methodology, Validation, Software. HX: Investigation, Methodology, Writing – review & editing, Software. SJ: Conceptualization, Investigation, Methodology, Supervision, Writing – review & editing, Funding acquisition, Resources, Project administration, Validation. LS: Conceptualization, Investigation, Supervision, Writing – review & editing, Funding acquisition, Resources, Project administration.
