# Supplementary material for: Paralichthys olivaceus MLKL-mediated necroptosis is activated by RIPK1/3 and involved in anti-microbial immunity
Source: Front Immunol. 2024 Jan 16;15:1348866. doi: 10.3389/fimmu.2024.1348866 (PMC10825024; doi:10.3389/fimmu.2024.1348866)

**Supplemental data**

**Table S1. Primers used in this study.**

| Primer | Sequence (5’-3’) |
| --- | --- |
| Primers for gene cloning  *PoRIPK1* forward  *PoRIPK1* reverse  *PoRIPK3* forward  *PoRIPK3* reverse  *PoMLKL* forward  *PoMLKL* reverse  Primers for protein overexpression  PoRIPK1-FL forward  PoRIPK1-FL reverse  PoRIPK1-ΔCT forward  PoRIPK1-ΔCT reverse  PoRIPK3-FL forward  PoRIPK3-FL reverse  PoRIPK3-K46A forward  PoRIPK3-K46A reverse  PoRIPK3-D146N- forward  PoRIPK3-D146N- reverse  PoRIPK3-S231A-S232A- forward  PoRIPK3-S231A-S232A- reverse  PoRIPK3-VQSG-AAAA- forward  PoRIPK3-VQSG-AAAA- reverse  PoMLKL-FL forward  PoMLKL-FL reverse  PoMLKL-4HB forward  PoMLKL-4HB reverse  PoMLKL-4HB-BR forward  PoMLKL-4HB-BR reverse  PoMLKL-T360E forward  PoMLKL-T360E reverse  PoMLKL-S361E forward  PoMLKL-S361E reverse  PoMLKL-S360A forward  PoMLKL-S360A reverse  PoMLKL-S361A forward  PoMLKL-S361A reverse  PoMLKL-T360E-S361E forward  PoMLKL-T360E-S361E reverse  PoMLKL-T360A-S361A forward  PoMLKL-T360A-S361A reverse  PoMLKL-G318D forward  PoMLKL-G318D reverse  PoMLKL-N105A-D106A forward  PoMLKL-N105A-D106A reverse  PoMLKL-K231D forward  PoMLKL-K231D reverse  PoMLKL-L293P forward  PoMLKL-L293P reverse  BFP-PoMLKL forward  BFP-PoMLKL reverse | ATGGCCACCGCGCCTCAGCC  TGAGATTGGGGAAGACAAAC  ATGGCTCTGTCCAGCTGCTC  TTGTTCTTCCAGCTCCATG  ATGGACTTTATAGATCCCA  CTCCTCAATCTGTGTCATC  CTACCGGACTCAGATCTCGAGATGGCCAC  CGCGCCTCAGCC  CGACTGCAGAATTCGAAGCTTGTTTGTCT  TCCCCAATCTCA  CTACCGGACTCAGATCTCGAGATGGCCAC  CGCGCCTCAGCC  GTGGTGGTGGTGGTGCTCGAGAGACCCT  GGCTCTTGTGGCA  TGAACCGTCAGATCCGCTAGCATGGCTCT  GTCCAGCTGCT  CGACTGCAGAATTCGAAGCTTTTTGTTCT  TCCAGCTCCA  GCTACGACATCGCCATTGCGCTGCTTCAT  TAC  GCAATGGCGATGTCGTAGCACCACTGACG  AT  CCGCCGTTCTCCACCTGAACCTGAAGCCC  AGC  TTCAGGTGGAGAACGGCGGGGGAGAGGC  TGTGG  TTACGCACAGGCAAAAGCCGCCATAGTG  CGGT  GCGGCTTTTGCCTGTGCGTAAGGTTGTTT  CC  AGCAACGTGACTGGAGCCGCCGCCGCCA  ACGAGAACA  GCGGCGGCGGCTCCAGTCACGTTGCTGT  AGGTGAT  CTACCGGACTCAGATCTCGAGATGGACTT  TATAGATCCCA  CGACTGCAGAATTCGAAGCTTCTCCTCAA  TCTGTGTCA  CTACCGGACTCAGATCTCGAGATGGACTT  TATAGATCCCA  CGACTGCAGAATTCGAAGCTTTTCAAAC  ACTTGGTACAGC  CTACCGGACTCAGATCTCGAGATGGACTT  TATAGATCCCA  CGACTGCAGAATTCGAAGCTTCACAAGC  TTTTCCACATTG  ACTGGCAAGAACAGAGGAGTCACTGAGA  AAGCCA  TCCTCTGTTCTTGCCAGTTCAAAACCTCC  CAGC  ACTGGCAAGAACAGAGACGGAACTGAGA  AAGCCA  TCCGTCTCTGTTCTTGCCAGTTCAAAACC  TCCCAGC  ACTGGCAAGAACAGAGGCGTCACTGAGA  AAGCCA  TCCGTCTCTGTTCTTGCCAGTTCAAAACC  TCCCAGC  ACTGGCAAGAACAGAGACGGCCCTGAGA  AAGCCA  GGCCGTCTCTGTTCTTGCCAGTTCAAAAC  CTCC  AACTGGCAAGAACAGAGGACGACCTGAG  AAAGCCA  GTCGTCCTCTGTTCTTGCCAGTTCAAAAC  CTCCC  AACTGGCAAGAACAGAGGCGGCGCTGAG  AAAGCCA  CGCCGCCTCTGTTCTTGCCAGTTCAAAAC  CTCCC  TGTGTCTGGACGCAGCACAAGACCTCTAC  CGATT  GTCTTGTGCTGCGTCCAGACACATACGAA  CTTT  CAGTGTGAATGAGCGCCTCGCTGCTGCCT  TCCAGCT  AGCAGCGAGGCGCTCATTCACACTGTTGA  ATTCGTCTC  TGGATTCACAGTGGTCATCGATAGATATAC  AGAC  ATCGATGACCACTGTGAATCCATTATACTC  TCCT  TACTGTGAGAAGGGGAGTCCGCGAGAGG  TTCTG  CGGACTCCCCTTCTCACAGTACTCCATGA  TAATCA  GGACGAGCTGTACAAGGGATCCATGGAC  TTTATAGATCCCA  AGTTCTAGACTCGAGAGATCTGTTACTCC  TCAATCTGTGTCA |

**Figure S1. Cytotoxicity of NSA and GW806742X.** HEK293T cells were incubated with different concentrations of NSA and GW806742X or equal volume of sterile PBS buffer (control) for 24 h. The release of LDH was measured.


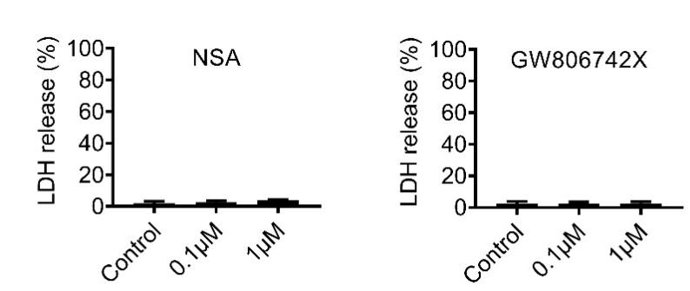


**Figure S2. Knock-down of PoMLKL by siRNA interference.** FG-9307 cells were transfected with or without (control) siRNA-MLKL or siRNA-NC for 24 h, and the expression of PoMLKL was determined by qRT-PCR. The expression level of PoMLKL in control cells was set as 1. Values are the means ± SD. n = 3. ***P* < 0.01


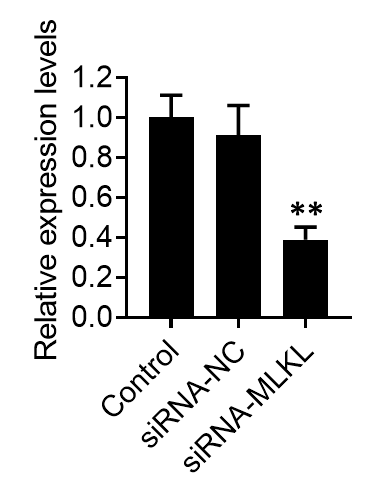

Supplement: Supplementary file 1 [file DataSheet_1.docx]
